# Using relational ethics to approach equity in palliative care

**DOI:** 10.1177/26323524241293820

**Published:** 2024-11-07

**Authors:** Kristina A. Smith, Kelli Stajduhar

**Affiliations:** Institute on Aging & Lifelong Health, University of Victoria, 3800 Finnerty Road, Victoria, BC V8P 5C2, Canada; Institute on Aging & Lifelong Health, University of Victoria, Victoria, BC, Canada

**Keywords:** decision-making, equity, ethics, palliative care, relational ethics, structural vulnerability

## Abstract

Evidence suggests that people experiencing inequities and who are highly marginalized (e.g., people impacted by racism, sexism, discrimination, stigma, mental illness, substance use issues, disability, and the effects of homelessness; also referred to as structurally vulnerable individuals) often die alone, in pain, and not receiving the care they need. Some research even points to highly marginalized people not feeling worthy of care. The need to consider equity in the context of palliative care has recently emerged but little attention has been paid to how ethical decision-making generally, and relational ethics, specifically, could provide guidance in the care of highly marginalized people who are on a palliative trajectory. Relational ethics offers a model of care and decision-making framework that emphasizes how clients, healthcare providers, and larger social structures are interwoven and acknowledge that structural conditions can position people to have less choice than others. Relational approaches in the context of palliative care for highly marginalized people have the potential to provide a lens to better support the delivery of equitable palliative care. This critical essay explores relational ethics as a way to approach equity in palliative care to support clients facing structural vulnerabilities. We discuss relational ethical considerations to approach collaborative partnerships between clients, healthcare providers, and the larger community with the goal of aligning care with clients’ values. An ethical case for how a relational ethics approach might be used to promote equitable access to palliative care will be explored, highlighting how such approaches have the potential to better align client wishes with their needs and to ensure decision-making and care delivery is trauma-informed, harm reduction focused, and culturally respectful. Relational ethics can support social change in equity and palliative care by contributing ethically informed ways of caring for/with/about highly marginalized people.

## Introduction

Everyone matters equally, but not everyone may be treated the same. Evidence from the extant literature highlights that people experiencing inequities and who are highly marginalized (hereby referred to as “structurally vulnerable”), including people impacted by poverty, racism, sexism, discrimination, stigma, mental illness, and/or substance use issues, disability, and the effects of homelessness more often die prematurely, alone and in pain, and not receiving the care they needed, some even expressing that they do not feel worthy of asking for care.^1,2^ Structural vulnerability is described as factors leading to inequitable and often poor health outcomes stemming from one’s position within larger social, cultural, political, material/economic hierarchies, such as views/stories of unworthiness and material value.^3^ People facing structural vulnerability are at a higher risk for negative health outcomes and are dying at a higher rate from treatable conditions in part because they cannot get access to care and/or because current health systems in the Global North are not designed, and often do not align with the needs of people who are structurally vulnerable.^2^ For example, home support policies that limit/prevent community care in the context of substance use and structural limitations (requiring shared bathrooms, space for providers to sit) and health system assumptions that clients have caregivers, friends, and family to support their needs in between health visits. Palliative care is a case in point. Despite international calls to integrate equity into palliative care, current palliative care services fall short in this regard.^2,4,5^

There is overwhelming evidence that health conditions develop and are perpetuated from stigma, living in poverty, having no fixed address or unstable housing, having little and inconsistent access to food, and having minimal to no family or inconsistent support networks.^1–3,6–10^ Further, research shows that individuals who are facing these inequities engage with the healthcare system and can experience re-traumatization, re-colonization, and re-institutionalization by the health institutions they are seeking help from.^2,7–9^ These experiences along with other complex social factors can result in people not being identified as in need of palliative care, in addition to facing challenges in accessing it.^11^ Integrating equity into palliative care recognizes and addresses the specific individual needs of people who experience structural vulnerability and the contexts in which they live.

The multiple and intersecting complex social, cultural, and structural factors that influence access to, and delivery of palliative care for people who are structurally vulnerable have received increasing attention in the past decade, highlighting the importance of moving beyond individual-level analyses to consider broader frameworks that would enable more equity-informed approaches to care.^12,13^ For clinical practice and decision-making, moving beyond individual-level assessments to consider the broader contexts which shape the lives of people who are structurally vulnerable is required if equity is to be promoted as a core consideration for palliative care. That is, while focus on individual-level needs is important, it is not sufficient to address the larger socio-structural considerations that are needed to realize equity in palliative care services.

In this paper, we explore relational ethics as a framework to guide the integration of equity into clinical practice and decision-making in palliative care in ways that are trauma-informed (acknowledging and understanding life experiences and creating trusting and kind spaces to facilitate healing),^14^ harm reduction focused (reducing risks associated with substance use and sexual practices),^15^ and culturally respectful (approach all people with respectful inquiry, and without judgment, of their unique identity, culture, worldview, lived experiences, and way of being).^16^ We begin with an overview of relational ethics and then, based on our collective research and clinical practice, provide a case example to show how a relational ethics approach can function practically between clients who are structurally vulnerable, healthcare providers (HCPs), and the community-based services and healthcare systems that support them. Building on our case example, we explore the importance of understanding how clients’ social circumstances, background, resources available to them, and current wishes and priorities impact decision-making and how a more holistic approach can serve those who experience structural vulnerability. We demonstrate a need to consider relational ethics as an approach to guide understanding of the relationality of care and suffering in people positioned as structurally vulnerable while at the same time providing palliative care clinicians with a framework to inform equity-informed clinical practice and decision-making in palliative care.

## Relational ethics

Relational ethics is a model of care and decision-making framework that emphasizes how clients, HCPs, and larger social structures are interwoven,^17,18^ and, acknowledges the structural conditions that position certain people to have less choice than others. In the context of making health and social care recommendations and decisions with clients, this framework encourages HCPs to understand clients’ values and needs in relation to the broader social contexts in which they live. In this way, relational ethics moves away from clients’ individual agency in making choices and moves toward understanding clients’ interdependence with the world around them. Decision-making is identified as a nonmaterial good that is essential for justice^19^ and Fraser^20^ centers justice in relationships, including material, cultural, and social. The potential contribution of relational ethics as an approach to care to enhance relationships, reciprocity, trust, and safety between clients and HCPs, as well as to support decision-making processes of HCPs is well documented within the field of bioethics.^17,18,21–24^ Less is written about how relational ethics aligns with health equity and the broader principles of palliative care with its focus on a holistic person and family-centered approach.^17,18^

**Figure 1.**
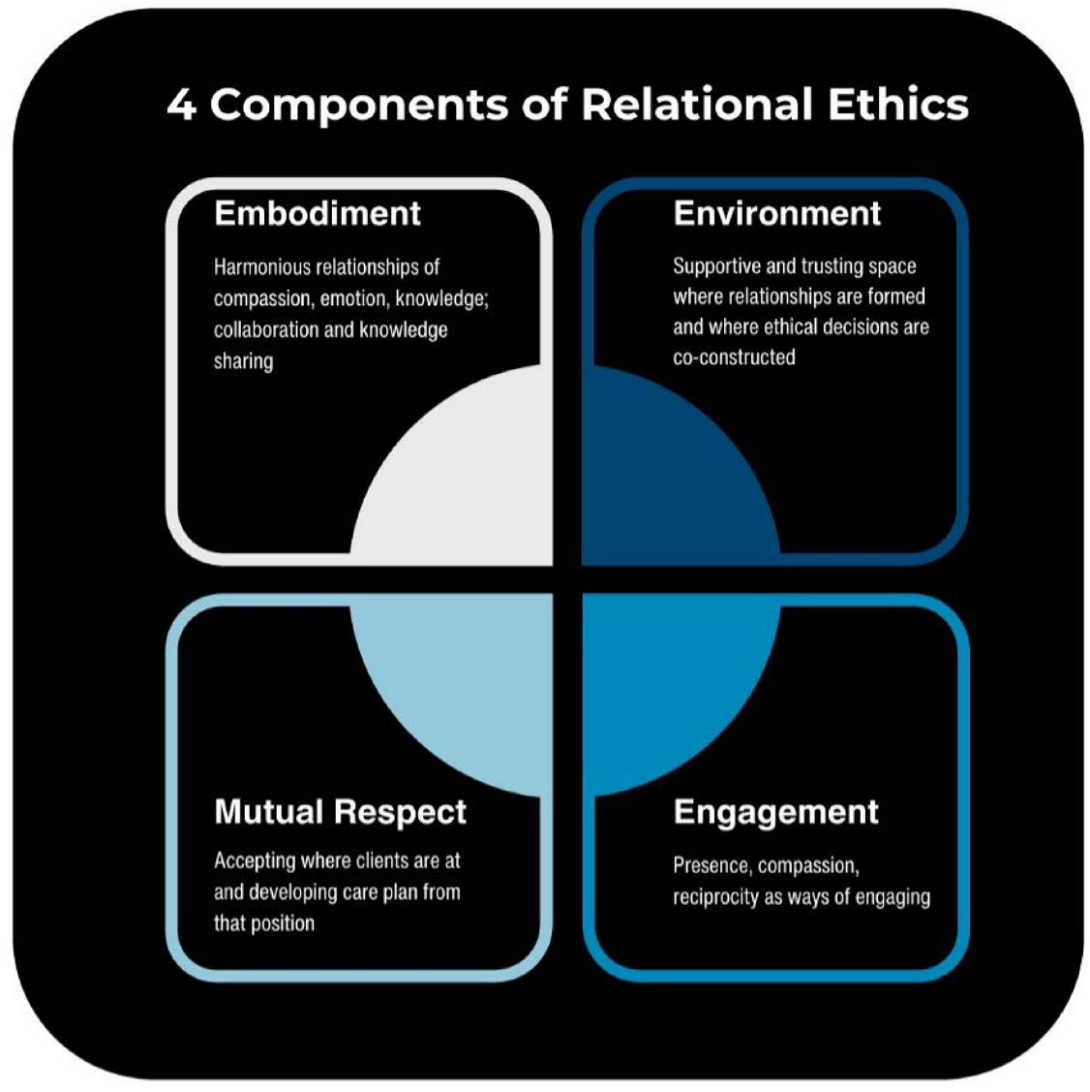
Four components of relational ethics. Source: Created and adapted from Austin et al.^21^, Olmstead et al.^22^ and Smith^23^

Austin et al.^21^ delineated four key components of relational ethics, embodiment, engagement, mutual respect, and environment. Olmstead et al.^22^ and Smith^23^ further expanded upon this in the context of pain and suffering, and we have further adapted and built upon these components, the first of which is *embodiment*, or the notion of acknowledging the lived experiences, stories, knowledge, and feelings of and held within other’s bodies, being attentive and attuned to one’s own emotional, physical, and intuitive states, as well as having harmonious relationships of compassion (collaboration and knowledge sharing). The second component is *engagement* and involves the ways in which we engage including presence, compassion, and reciprocity. The third component is *mutual respect*, which involves accepting “where clients are at” and developing care plans from that position. The fourth and final component is *environment*. This entails a supportive and trusting space where relationships are formed. Bergum and Dosseter^18^ express the need for a relational space to negotiate ethical decisions for “as we ‘feel’ the other person in this common space, it is difficult to be immune to the effects our actions have on the other.”^22^ A relational space is a physical, mental, emotional, and interactive field shared between people that affords the co-construction of knowledge between people (Figure 1).

Relational ethics represents a unique interactive framework for clinical care and decision-making that establishes relationships as the foundation of care; it takes the position that care and decision-making recognize that people in the care encounter (e.g., clients and HCPs) are interconnected, co-constructing, and trying to make sense of sometimes complex and uncertain clinical and social processes. One way to *see* a relational ethics approach is by using the analogy of a relational web^23^ which was inspired by Hartman’s^25^ ecological maps—to show individual or family systems. A client is in the center of the web with numerous people, services, and structures surrounding them such as cultures, family, HCPs, resources available, health organizations, media, ethics, and laws. This web can highlight the geographies of people’s lives and importantly, illustrate the source of numerous and complex belief systems and deeply held values of clients. Clients do not come into a hospital or into contact with an HCP solely as individuals to be treated, but as a part of a collective^26^; this means that clients are interdependent and interconnected with particular people and relationships, values, and beliefs, class structures, cultural backgrounds, religious and/or spiritual practices, among a range of other social processes that HCPs are working with and within throughout clients’ care. Therefore, a relational web can provide a visual of the many people, structures, and systems that shape the values, beliefs, and wishes clients hold and express and the multidimensional perspectives that HCPs are encouraged to understand and consider in their ethically informed care recommendations. To illustrate, we offer a case example to show how key principles of relational ethics can be applied to promote equity in palliative care.

## Sherry: A case example of a relational ethics approach to care

Sherry, a 59-year-old woman, has terminal diagnosis, cognitive impairment, a trauma history, and uses illicit substances. She is approaching end-of-life and uses hospice services but regularly leaves, sometimes for a few days, to use illicit substances and visit her father and partner. She is presumed capable of healthcare, personal care (e.g., housing), and financial decisions. Recently, she was upset after the hospice discharged her after not returning for several days. She would like to keep her bed at the hospice but does not want to stay full-time. The Most Responsible Provider (a regulated HCP who has overall responsibility for coordinating and providing care for a client) worries that Sherry will severely decline in the community, and the team is morally distressed (the discomfort caused by the inability to act in accordance with one’s beliefs)^27,28^ over how to best provide care due to her unpredictable returns. The team is considering holding Sherry’s bed for her but are concerned about resource fairness for other clients (e.g., beds available).

We discuss the application of the four components of relational ethics and our recommendations for Sherry’s case under three subheadings: engagement, mutual respect, and embodiment and environment.

### Engagement

Relational ethics, and specifically, the component of *engagement*^21,22^ looks at the process of how HCPs engage with Sherry and her relational web—all the relationships and structural conditions impacting Sherry’s web from a trauma-informed and culturally respectful lens. Sherry is marginalized and experiencing multiple, intersecting inequities such as past trauma, cognitive impairment, illicit substance use, and precarious housing, and has a complex relationship with her father and partner who are both significant influencers on her decision-making. Also in her web are the hospice staff and numerous other HCPs and health institutions that she has interacted with that may or may not contribute to re-traumatization. Sherry’s wish to receive care from the hospice, albeit in a different form than is typically offered and resource allocation constraints are in tension. Sherry wants to have flexibility in her hospice care and be able to leave to visit her family/chosen family in the community^3^ where she feels at home and supported but also desires the security of a bed. This flexibility may enhance Sherry’s quality of life and promote the quality of palliative care as her health declines but her values and wishes are in conflict with what can be allocated fairly (distributive justice; e.g., staff to have “eyes on her” in the community, beds). Ensuring the fair distribution of benefits and harms, and risks and burdens, when resources are limited is important but can be in tension with what health systems also value. Ethically and trauma-informed, equitable distribution of resources facilitates not placing unfair burdens on a particular person/group and not perpetuating systemic or structural inequities (e.g., geographic obstacles that create barriers to accessing resources and/or social policies or processes) and should attempt to improve inequities, where possible. Further, ethics values flexibility and beginning with the least intrusive intervention. Given her relational web and background and her expressed wish for care from the hospice at this time, from a relational ethics perspective, there is a higher obligation to accommodate and provide Sherry with a different approach which might mean trialing, as a first step, keeping a bed open for her. Staff also need to engage with Sherry to better understand her evolving needs, how much time she would like to spend out of the hospice, and have ongoing conversations with Sherry about her preferences for care (does she still want to be in hospice, want to be engaging with her family in the community, would Sherry like her family visit more often at the hospice). Sherry may be more comfortable having these conversations in particular environments such as the hospice, community, or with or without her family, and therefore, documenting Sherry’s preferences in her care plan and regularly re-visiting these with Sherry is important as her wishes and preferences may change due to the precarity and vulnerability of her living conditions and the complexities of her relational web. This approach honors intersubjectivity,^29^ or a *reciprocal union*^23^ between clients and their HCPs in which accommodations and adjustments are made from a shared understanding of the need and curious, open, and kind ongoing exploration of those needs.

### Mutual respect

The component of *mutual respect*^21,22^ enables exploring “where clients are at” and in the process being open to trialing new, and less traditional approaches, especially when current care plans are not aligning with the clients’ care needs. Apart from trialing new approaches, mutual respect encompasses setting boundaries for decisions made with kindness and compassion within the accommodations made. This might mean setting a limit on the number of days Sherry can be away from the hospice and setting some check-in times with the hospice staff. Such accommodations will require consideration of whether Sherry can follow through on these limits given her mental health and substance use. Realistic goals would need to be set to ensure success for Sherry. Relational ethics facilitates consistent reassessment by taking a holistic picture of Sherry’s needs, values, and relational web and the risks of harm with different care pathways. For example, without access to a hospice bed, Sherry will likely decline in the community and be brought to the emergency department, risking further perpetuation of past traumas. From this example, we highlight that the harms of not having a bed available are more certain and known than the harms of maintaining a bed for Sherry, and thus, leaving a bed open offers a culturally respectful (aligning with her values, ways of being), harm reducing (reducing risks of severe decline), and trauma-informed care pathway.

A part of the holistic assessment of Sherry would also include the acknowledgment that the structural conditions that she lives in—such as poverty, housing precarity, and few caregivers to support her needs—can facilitate more vulnerability (e.g., unmanaged pain (and increased substance use for pain relief), access to medication, suffering, lack of food and water, loneliness) when approaching the end of her life.^3,11^ Supporting people who experience structural vulnerabilities requires palliative care providers to think about who the care networks are, and whether they are able to provide the support needed, and therefore, palliative care may need to draw on *engaging* and *developing mutual respect* with the communities and workers that provide the vast majority of support. With respect to Sherry, this may look like the hospice and community teams coordinating services and working in partnership to support Sherry in maintaining her care (e.g., medication management and adjusting based on substances used in the community) and responding if her health declines (e.g., support her in getting to hospice).

### Embodiment and environment

Within this subsection, we discuss the components of embodiment and environment together because they are inherently linked. We show that the compassionate relationships felt (embodied) knowledge and collaborations formed can set up the space of trust and ethical decision-making in the environmental component. The concept of embodiment focuses on cultivating a coordinated approach informed by different ways of knowing (felt, lived experience, and shared knowledge) to, and continuity of, care. Leading scholars in equity and palliative care highlight the importance of building connections within healthcare and the need for a more collaborative approach as a way to break down the common silos between teams and health organizations and to better coordinate services.^6–9^ Reimer-Kirkham et al.^8^ explore various gaps in care for clients experiencing structural vulnerability at end-of-life among palliative care and inner-city service providers and highlight that HCPs acknowledge the disconnect in care between HCPs in homecare and acute hospital settings (e.g., emergency departments) and community teams who are often providing informal palliative care to clients experiencing structural vulnerability. Brown et al.^6^ show that to enhance equity-oriented primary care at the macro (health organizations), meso (clinical programming), and micro (client-provider interactions) levels, care must be coordinated, contextually and culturally tailored and trauma-informed to foster trust between clients and shift away from crisis-oriented approaches to care. Relational ethics contributes to equity in palliative care for the structurally vulnerable because it situates ethical practice *in* relationships, which allows the transparent and compassionate exploration of options and the values upheld within each option *in relation* to the client and HCPs (micro), hospice/community/acute services (meso), and health organizations/government (macro). Further, relational ethics promotes interdependence—a reciprocal union—rather than independence between relationships and structures. This approach, then, enables HCPs to see the client and their in-depth needs and the possible routes to collaborating with others to provide continuity, for example, building stronger involvement with peer support workers (people who identify as having lived/living experience and provide support to their peers).^30^ In this way, relational ethics brings a unique focus on social relations and structures to support, both, looking at individual-level and greater social and collectives forces that equity and palliative scholars are asking for more attention to be given.^8^

The lack of continuity in some healthcare services creates significant barriers for clients who are structurally vulnerable,^9^ which can also significantly impact the *environment* of care for clients. Therefore, trust and consistency are essential to filling these gaps in care and to facilitate shared ethical decision-making.^9^ By exploring Sherry’s web with a relational ethics approach, flexible and adaptable options such as trialing a higher level of accommodation—leaving a bed open—and stronger collaboration between teams/services such as hospice, community, and the emergency department may outweigh the resource constraints. Regardless of the care plan, all teams need to be on board and consistent with the approach to care, and clear on the values that each option upholds, which further highlights the importance of breaking down silos and collaborative care planning.

## Moral distress

In this paper, we have shown that relational ethics can provide a framework for decision-making to achieve equity in palliative care. Relational ethics can also help with the moral distress that is reported among HCPs working with clients experiencing structural vulnerability.^24^ Within Sherry’s case, some of the HCPs expressed feeling moral distress over not being able to provide the quality of care they would like to Sherry given external factors such as resources and Sherry’s preferences and wishes. Decision-making in the face of resource constraints and end-of-life care issues is a significant threat to moral integrity and can lead to moral distress (unable to uphold values and/or act on patient’s behalf due to internal or external constraints)^27,28^; and moral injury (repeated incidence/persistent feelings of moral distress/deeply felt violation to one’s beliefs).^28,31^ Moral distress and injury are particularly high in contexts where HCPs are placed in positions of making life-or-death decisions when they are working with adults who are structurally vulnerable.^27,31^ This moral distress can result in moral injury and deepen and lengthen the effects of distress, impacting the physical, social, emotional, mental, and spiritual dimensions of HCPs’ lives. Clients are also affected by moral injury in HCPs as resentments, anxiety, burnout, dissatisfaction at work and other symptoms bleed into care and/or by loss of staff.^27,31,32^ In this way, moral distress and injury can influence HCP’s capacity to care for others. Although a relational ethics approach may not fully remove the moral distress that some HCPs may feel in situations such as Sherry’s case, it can expand HCP’s lens of clients and facilitate seeing their web and the structural conditions that may be affecting Sherry’s values, preferences, and wishes. With an expanded view and a diversity of partnerships/perspectives, more options become available to try and multiple teams can engage in collaborative care planning rather than these plans resting with solely one team. It is the process of creatively trialing all possible options—or providing a justification of why certain options cannot be tried—and reciprocal engagements that may involve validation, support, access to resources, and idea generation, which can help reduce moral distress.

## Conclusion

A relational ethics approach to equity in palliative care explores the relationships and larger structures that affect clients’ values and beliefs as well as those that limit the choices people have. The four components of relational ethics are interdependent, providing a guide for how HCPs engage with each other and with clients to develop mutual respect and embodied ways of relating which ultimately facilitates the process of building a trusting environment for care. Therefore, each component evolves as the needs of client’s change and shift. At the heart of relational ethics is respect, reciprocity, dignity, connection, and shared knowledge around how HCPs and health systems engage with each other and their clients alongside their relational webs and current situations (“what is”), and as such, it supports both individuals and collective focuses within equity and palliative care. These individual and collective lenses complement the holistic approach to care that is foundational in palliative care. Further engagement with relational ethical approaches will provide more opportunity to explore what is needed to bridge connections between and within health institutions and teams in reciprocal unions such as hospice and community care teams and to explore hybrid (shared) models of care. We acknowledge that there can be challenges faced by HCPs in adopting a relational ethics approach such as ensuring continuity of care from hospice to community services and balancing individual client needs within resource-constrained settings. Providing continuity of care between teams requires HCP, leadership, and organizational buy-in, and time and energy to form collaborative approaches, one effective strategy being holding interdisciplinary case conferences (can include client, all supporting teams, and ethics). Additionally, settings working within resource constraints like hospice and community sites may not always be able to uphold a client’s wishes and preferences if it may create harm to others such as harm to those on a waitlist for a hospice bed. If a waitlist forms for beds at the hospice and is made up of clients with equal or more complex clinical needs, and Sherry wants to be out in the community for 3–4 days a time/leaves the hospice for days at a time without communicating when she may be back, then it is possible that the resource needs of others may impact Sherry’s preferences. This brings us back to the importance of ongoing assessments of clients’ needs and exploring creative options. Teams can explore contingency plans that may offer Sherry respite stays with daypasses rather than a full-time bed at the hospice, and explore if a palliative care unit at the hospital could meet her needs in the future (mindful of institutional trauma experienced) as well as when that threshold may be, and how her pain can be managed when she stays in community for longer periods of time. In this way, ethical practice, specifically relational ethics, involves exploring and trialing all possible options, ongoing assessments of client’s needs, and expanding collaborations to provide the best possible care based on what is needed at that moment.

## Declarations

**Ethics approval and consent to participate:** Ethical approval was not required for this critical essay as it does not include data/research. Consent to participate: Not applicable.

**Consent for publication:** Not applicable.

**Availability of data and material:** Not applicable.

## References

[1] Baggett TP Hwang SW O’Connell JJ , et al. Mortality among homeless adults in Boston: shifts in causes of death over a 15-year period. JAMA Intern Med 2013; 173(3): 189–195. 23318302 10.1001/jamainternmed.2013.1604 PMC3713619

[2] Stajduhar KI Mollison A Gleave D , et al. When cancer hits the streets. Curr Oncol 2017; 24(3): 149–150. 28680272 10.3747/co.24.3698 PMC5486377

[3] Stajduhar KI Giesbrecht M Mollison A , et al. Caregiving at the margins: an ethnographic exploration of family caregivers experiences providing care for structurally vulnerable populations at the end-of-life. Palliat Med 2020; 34(7): 946–953. 32340556 10.1177/0269216320917875 PMC7787672

[4] Stajduhar K Gott M . Closing the health equity gap in palliative care: the time for action is now. Palliat Med 2023; 37(4): 424–425. 37010057 10.1177/02692163231164729

[5] Connor SR Gwyther E . The worldwide hospice palliative care alliance. J Pain Symptom Manag 2018; 55(2): S112–S116. 10.1016/j.jpainsymman.2017.03.020 28797861

[6] Browne AJ Varcoe CM Wong ST , et al. Closing the health equity gap: evidence-based strategies for primary healthcare organizations. Int J Equity Health 2012; 11: 1–15. 23061433 10.1186/1475-9276-11-59 PMC3570279

[7] Lett E Adekunle D McMurray P , et al. Health equity tourism: ravaging the justice landscape. J Med Syst 2022; 46(3): 17. 35150324 10.1007/s10916-022-01803-5 PMC8853313

[8] Reimer-Kirkham S Stajduhar K Pauly B , et al. Death is a social justice issue. Adv Nurs Sci 2016; 39(4): 293–307. 10.1097/ANS.0000000000000146 27608146

[9] Stajduhar KI Mollison A . Too little, too late: how we fail vulnerable Canadians as they die and what to do about it. Report, University of Victoria Institute on Aging and Lifelong Health, Canada, November 2018.

[10] World Health Organization. Closing the gap in a generation: health equity through action on the social determinants of health. Report, Commission on Social Determinants of Health, Switzerland, 2008. 10.1016/S0140-6736(08)61690-6 18994664

[11] Stajduhar KI Mollison A Giesbrecht M . et al. “*Just too busy living in the moment and surviving*”: barriers to accessing health care for structurally vulnerable populations at end-of-life. BMC Palliat Care 2019; 18: 11. DOI: 10.1186/s12904-019-0396-7. 30684959 PMC6348076

[12] Richards N . The equity turn in palliative and end of life care research: lessons from the poverty literature. Sociol Compass 2022; 16(5): e12969.

[13] Stajduhar KI . Provocations on privilege in palliative care: are we meeting our core mandate? Prog Palliat Care 2020; 28(2): 89–93. DOI: 10.1080/09699260.2019.1702334.

[14] British Columbia Provincial Mental Health and Substance Use Planning Council. Trauma-informed practice guide. Report, BC Centre of Excellence for Women’s Health & BC Ministry of Health, Mental Health and Substance Use Branch, Canada, http://bccewh.bc.ca/wp-content/uploads/2012/05/2013_TIP-Guide.pdf (2013, accessed 22 June 2024).

[15] Vancouver Coastal Health. Harm reduction, https://www.vch.ca/en/health-topics/harm-reduction (2024, accessed 22 June 2024).

[16] Virani A Everett B Adurogbangba M , et al. Ethical analysis of prescribed safer supply policy in British Columbia: final report and recommendations. In: A review of prescribed safer supply programs across British Columbia: Recommendations for future action. Report, Office of the Provincial Health Officer, Canada, pp.62–96, https://www2.gov.bc.ca/assets/gov/health/about-bc-s-health-care-system/office-of-the-provincial-health-officer/reports-publications/special-reports/a-review-of-prescribed-safer-supply-programs-across-bc.pdf (2023, accessed 22 June 2024).

[17] Bergum V . Relational ethics for health care. In: Storch J Rodney P Starzomski R (eds) Toward a moral horizon: nursing ethics in leadership and practice. 2nd ed. Toronto: Pearson Education Canada, 2012, pp.127–142.

[18] Bergum V Dossetor JB . Relational ethics: the full meaning of respect. Hagerstown: University Publishing Group, 2005.

[19] Young IM . Justice and the politics of difference. Princeton: Princeton University Press, 2011.

[20] Fraser N . Justice in the age of identity politics: redistribution, recognition, and participation. Lecture, Social Science Research Center, Germany, https://www.econstor.eu/bitstream/10419/44061/1/269802959.pdf (1998, accessed 10 April 2024).

[21] Austin W Bergum V Dossetor J . Relational ethics: an action ethic as a foundation for health care. In: Tshudin V (ed.) Approaches to ethics. Woburn: Butterworth-Heinemann, 2003, pp.45–52.

[22] Olmstead DL Scott SD Austin WJ . Unresolved pain in children: a relational ethics perspective. Nurs Ethics 2010; 17(6): 695–704. 21097968 10.1177/0969733010378932

[23] Smith K . Exploring waiting for child transplant candidates and families. PhD Thesis, University of Toronto, Canada, 2022.

[24] Hodgetts D Rua M Groot S , et al. Relational ethics meets principled practice in community research engagements to understand and address homelessness. J Commun Psychol 2022; 50(4): 1980–1992. 10.1002/jcop.22586 33999450

[25] Hartman A . Diagrammatic assessment of family relationships. Soc Casework 1978; 59(8): 465–476.

[26] Mol A . The logic of care: health and the problem of patient choice. New York: Routledge, 2008.

[27] Jaskela S Guichon J Page SA , et al. Social workers’ experience of moral distress. Can Soc Work Rev 2018; 35(1): 91–107.

[28] Kok N Hoedemaekers A van der Hoeven H , et al. Recognizing and supporting morally injured ICU professionals during the COVID-19 pandemic. Intensive Care Med 2020; 46(8): 1653–1654. 32468083 10.1007/s00134-020-06121-3 PMC8824542

[29] Csordas T . Intersubjectivity and intercorporality. Subjectivity 2008; 22(1): 110–121.

[30] Lennox R Lamarche L O’Shea T . Peer support workers as a bridge: a qualitative study exploring the role of peer support workers in the care of people who use drugs during and after hospitalization. Harm Reduct J 2021; 18: 1–9. 33593364 10.1186/s12954-021-00467-7 PMC7885412

[31] Corley MC . Nurse moral distress: a proposed theory and research agenda. Nurs Ethics 2022; 9(6): 636–650. 10.1191/0969733002ne557oa 12450000

[32] DeBoer RJ Mutoniwase E Nguyen C , et al. Moral distress and resilience associated with cancer care priority setting in a resource-limited context. Oncologist 2021; 26(7): e189–e1196. 10.1002/onco.13818 PMC8265342 33969927

